# Short stretches of rare codons regulate translation of the transcription factor ZEB2 in cancer cells

**DOI:** 10.1038/onc.2017.273

**Published:** 2017-08-07

**Authors:** W R Wan Makhtar, G Browne, A Karountzos, C Stevens, Y Alghamdi, A R Bottrill, S Mistry, E Smith, M Bushel, J H Pringle, A E Sayan, E Tulchinsky

**Affiliations:** 1 Department of Cancer Studies, University of Leicester, Leicester, UK; 2 MRC Toxicology Unit, Leicester, UK; 3 Protein and Nucleic Acid Chemistry Laboratory (PNACL), University of Leicester, Leicester, UK; 4 Cancer Sciences Division, University of Southampton, Southampton, UK

## Abstract

Two proteins comprising the ZEB family of zinc finger transcription factors, ZEB1 and ZEB2, execute EMT programs in embryonic development and cancer. By studying regulation of their expression, we describe a novel mechanism that limits ZEB2 protein synthesis. A protein motif located at the border of the SMAD-binding domain of ZEB2 protein induces ribosomal pausing and compromises protein synthesis. The function of this protein motif is dependent on stretches of rare codons, Leu(UUA)-Gly(GGU)-Val(GUA). Incorporation of these triplets in the homologous region of ZEB1 does not affect protein translation. Our data suggest that rare codons have a regulatory role only if they are present within appropriate protein structures. We speculate that pools of transfer RNA available for protein translation impact on the configuration of epithelial mesenchymal transition pathways in tumor cells.

## Introduction

Transcription factors belonging to several Zinc finger or bHLH protein families (SNAIL, ZEB, TWIST and so on) are downstream effectors in a number of signaling pathways driving reversible epithelial mesenchymal transition (EMT) programs in normal and pathological conditions. During the entire process of tumourigenesis, these transcription factors contribute to malignant transformation, invasion, regulate cancer cell stemness, drug resistance and metastasis.^1, 2^ The reversion of EMT, mesenchymal epithelial transition, is an essential step, when cancer cells colonize distant organs and establish secondary growth. Tumor cell plasticity or the ability to balance between epithelial and mesenchymal cell states is a key factor in metastatic process. ZEB1 and ZEB2 transcription factors and members of the microRNA-200 family form a double negative feedback loop that regulates EMT/mesenchymal epithelial transition balance.^3^ An interaction between ZEB/miR-200 network and a number of oncogenic and tumor suppressor pathways takes place at different stages of the metastatic cascade.^4^

ZEB proteins are large transcription factors featured by the presence of two separated clusters of zinc fingers binding to the promoter DNA of target genes. Interactions between ZEB proteins and components of different co-repressor and co-activator complexes are essential for ZEB-mediated transcriptional repression and activation. ZEB1 and ZEB2 exhibit a considerable level of sequence similarity, especially in zinc finger domains, suggesting that they interact with the same arrays of target promoters.^5^ However, emerging evidence suggests dissimilar and possibly even opposing roles for ZEB1 and ZEB2 in different cancer types. ZEB2 represses cyclin D1 or hTERT leading to the reversible cell cycle arrest or cellular senescence.^6, 7^ In cells of cutaneous melanoma, ZEB2 increases expression levels of a tumor suppressor protein PTEN^8^, and promotes melanocytic differentiation through upregulation of MITF.^9^ In contrast, ZEB1 does not possess these oncosuppressive functions. Moreover, its oncogenic, pro-proliferative and metastases-promoting features are well documented. In line with these data, ZEB1 and ZEB2 proteins have a potential of reciprocal negative regulation.^9, 10, 11^ Here, we show that ZEB2 expression is controlled at yet another level, protein synthesis. We propose that the underlying mechanism represents a novel regulatory scheme based on ribosome pausing and degradation of the nascent peptide.

## Results

### ZEB2 is downregulated at posttranscriptional level

**Figure 1:**
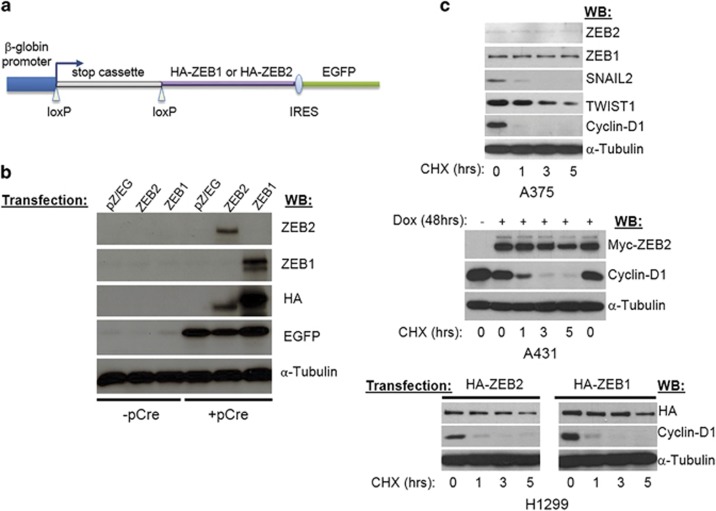
ZEB1 and ZEB2 are stable proteins differentially regulated via an unidentified mechanism. (**a**) A scheme of constructs used in the transfection experiments in (**b**) and Supplementary Figure S2. (**b**) HEK-239 cells were transfected with pZ/EG vectors co-expressing ZEB1 or ZEB2 with EGFP, or with the pZ/EG empty vector. pCre recombinase expression vector was included in some transfections as shown. Protein expression was analyzed by immunoblotting with indicated antibodies. (**c**) ZEB1 and ZEB2 are long-lived proteins. Melanoma A375 cells co-expressing ZEB1 and ZEB2 proteins, or carcinoma cell lines A431 and H1299 with ectopic expression of ZEB proteins were treated with cycloheximide and analyzed in immunoblotting.

Although ZEB1 protein is present in the majority of carcinoma cell lines exhibiting mesenchymal properties, expression of ZEB2 is uncommon. On the other hand, both gene transcripts are present in most of the mesenchymal cell lines^12^ (Supplementary Figure S1). We noticed that transfection of different cell cultures with the same expression vectors harboring either ZEB1 or ZEB2 ORFs resulted in a much higher ZEB1 expression and more evident EMT (data not shown). As ZEB2, but not ZEB1 was reported to promote quiescence or cellular senescence,^6, 7^ the observed phenomenon can be explained by the elimination of ZEB2-expressing cells from transfected cell cultures. Alternatively, ZEB2 expression can be restricted by presently unidentified molecular mechanisms acting at a posttranscriptional level. To discriminate between these hypotheses, we generated two pZ/EG plasmid-based vectors with cre recombinase-dependent expression of ZEB2 or ZEB1 proteins. Cre recombinase-based excision of a stop cassette activated expression of bi-cistronic messenger RNAs, in which internal ribosome entry sites separated ZEB2 or ZEB1 ORFs from EGFP (Figure 1a). In both constructs, ZEB protein ORFs were fused with HA tags; and the same *ZEB1*-derived Kozak element was used in both constructs to initiate translation of ZEB1 or ZEB2 proteins. Application of these vectors in transient transfections of different cell cultures allowed us to use EGFP as an internal control for comparison of ZEB1 and ZEB2 expression levels. Transfection of the HEK-293 cell line with these constructs indicated similar levels of cre-dependent expression of EGFP from both pZ/EG-HA-ZEB2, pZ/EG-HA-ZEB1 and from empty pZ/EG vector indicating that the transfection efficiency was unaffected by ZEB1/2 insertion (Figure 1b). On the other hand, expression level of ZEB2 was much lower than that of ZEB1 in HEK-293 (Figure 1b) and four other cell lines A431, MDA-468, H1299 and SaOs-2 (Supplementary Figure S2). This difference was especially evident in carcinoma cell lines retaining epithelial features, A431 and MDA-MB-468. The results of these experiments allowed us to conclude that: (i) ZEB2-expressing cells were not selectively eliminated from the transfected cell cultures; (ii) decreased level of ZEB2 was independent of microRNA (3’UTR sequences were not incorporated into the constructs), and was likely to represent a novel intrinsic feature of ZEB2 ORF.

EMT inducers SNAIL1, SNAIL2 and TWIST1 are short-lived proteins degraded via ubiquitin-proteasome pathway. ZEB proteins are regulated by analogous mechanism at the level of protein stability as previously reported.^13, 14^ However, cycloheximide chase experiments indicated that both ZEB1 and ZEB2 proteins are nearly equally stable in melanoma cells where both proteins are present. Likewise, both proteins ectopically expressed in cells of epithelial origin are stable, and the half-life of ZEB2 is independent of added tag sequences (Figure 1c). A half-life of either protein exceeded 5 h (Figure 1c). High stability of both ZEB proteins overexpressed in H1299 cells was confirmed in ^35^S-Met/Cys metabolic labeling experiments (data not shown).

### A region limiting ZEB2 expression level is adjacent to the SMAD-interacting domain

**Figure 2:**
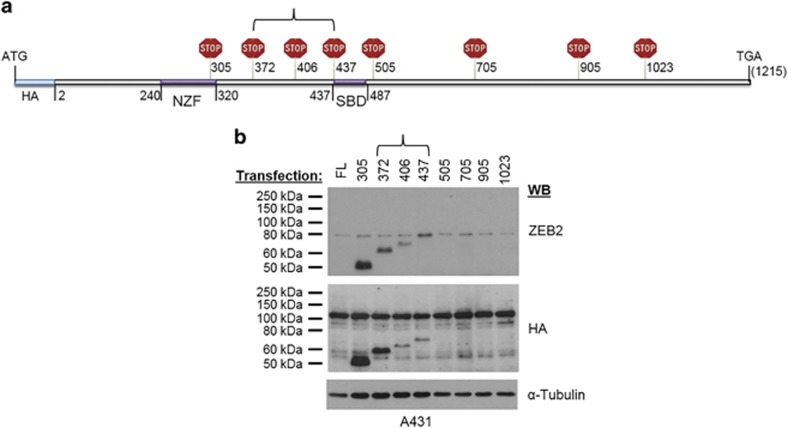
An element determining the reduced ZEB2 expression is adjacent to the SMAD-interacting domain. (**a**) A scheme of constructs producing truncated ZEB2 mutant proteins. Coordinates of the inserted stop codons are indicated. (**b**) Expression level of full-length (FL) or truncated ZEB2 protein was analyzed by immunoblotting in A431 cells transiently expressing indicated proteins.

**Figure 3:**
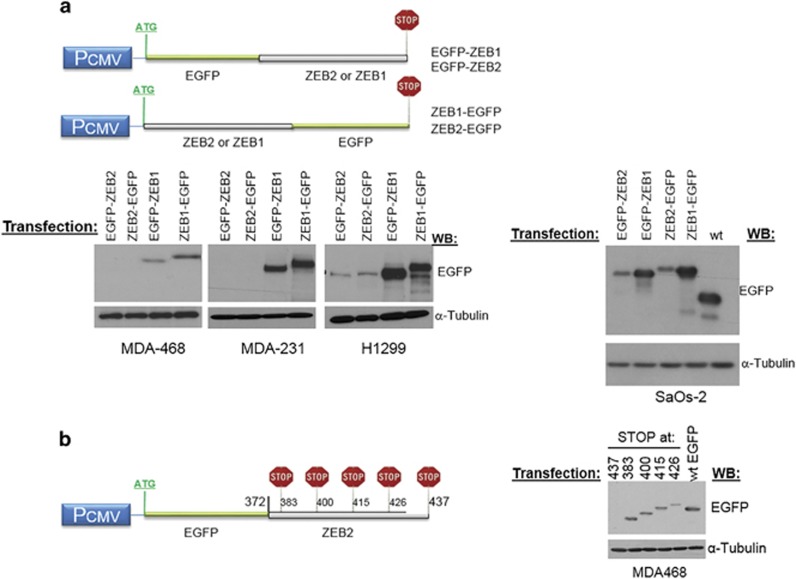
Precise mapping of the element that limits ZEB2 expression levels. (**a**) Fusion of EGFP with ZEB2-derived 372–437 aa fragment reduced expression of EGFP. This fragment or homologous 321–372 aa ZEB1 sequence (Supplementary Figure. S3) was fused with EGFP N- or C-terminally as shown in the scheme. Expression of the chimeric proteins was analyzed in transiently transfected cell lines by immunoblotting. (**b**) Stop codons were introduced in the EGFP-ZEB2 expression construct as shown. EGFP-ZEB2 expression was analyzed in MDA-468 cells.

Next, we aimed at identifying which element in ZEB2 protein structure is a determinant of its reduced expression in transfection experiments. To this end, we inserted STOP codons into ZEB2 ORF cloned in the pcDNA 3.1/TOPO-HA-ZEB2 expression construct (Figure 2a). ZEB2 expression was analyzed by immunoblotting of proteins isolated from cells transiently transfected with this series of constructs. Presence of the sequence located between amino acids 372 and 437 strongly reduced expression levels of *C*-terminal truncated mutants suggesting that this region may have a regulatory role (Figure 2b). A total of 372–437 region is adjacent to the SMAD-Binding Domain, SBD (Figure 2a) and includes an IKTE motif representing a sumoylation site.^15^ Alignment of this fragment in ZEB2 with the equivalent region in ZEB1 shows low conservation, indicating that the function of this region might be ZEB2-specific (Supplementary Figure S3). To test this hypothesis, we generated vectors expressing chimeric proteins, in which ZEB2 372–437 aa fragment was fused with EGFP either N- or C- terminally. As a control we used identical constructs but containing the corresponding ZEB1 fragment (310–372 aa) (Figure 3a). Fusion of the ZEB2, but not ZEB1 fragment almost completely inhibited EGFP expression in MDA-MB-468 and MDA-MB-231 cells (Figure 3a). In H1299 and SaOs-2 cells, the expression of ZEB2 fusion proteins was detectable, but much lower than that of EGFP-ZEB1 or ZEB1-EGFP.

To map a domain limiting the expression of ZEB2 fusion proteins more precisely, we generated a series of constructs by introducing translation stop codons into EGFP-ZEB2 expression vectors (Figure 3b). Transfection of these constructs in MDA-MB-468 cells demonstrated that the sequence encompassing the last 12 amino-acid residues was essential for maintaining the function of the 372–437 element. This also indicated that a potential sumoylation site (aa 390–393) (Supplementary Figure S3), had no impact on the ability of ZEB2-derived sequence to limit expression of the fusion protein.

### 372–437 aa sequence element destabilizes EGFP and induces ribosome stalling

**Figure 4:**
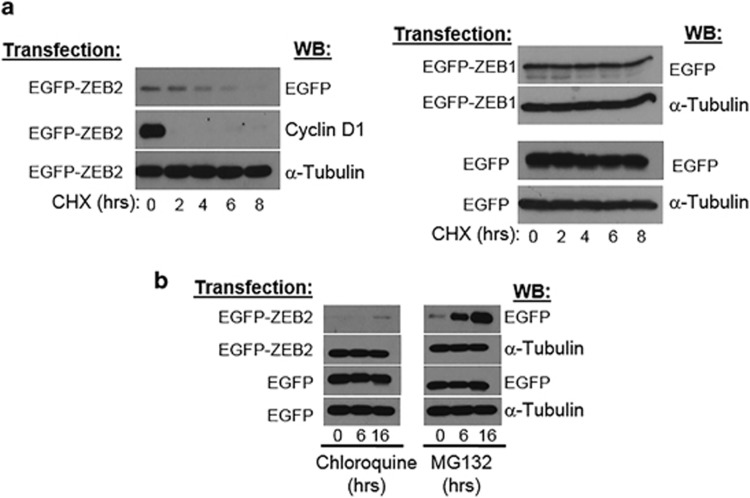
Fusion with the 372–437 aa ZEB2 fragment confers instability on EGFP. (**a**) EGFP-ZEB1, EGFP-ZEB2 or EGFP proteins were expressed in MDA-468 cells. After 48 h of transfection, cells were treated with cycloheximide (20 mg/ml) for indicated time periods, and analyzed in western blotting. A short-lived cyclin D1 protein was used as an internal control. (**b**) Proteasomal inhibitor MG132, but not lysosomal inhibitor, chloroquine increases the expression of EGFP-ZEB2 fusion. MDA-468 cells transiently transfected with the indicated constructs were treated with the inhibitors as shown, and the expression was analyzed using an anti-EGFP antibody.

Our next aim was to gain mechanistic insight into the function of the 372–437 aa element. Though the full-length ZEB2 was a long-lived protein (Figure 1c), fusion with the 372–437 aa fragment derived from ZEB2 protein conferred instability on EGFP-ZEB2. In contrast, destabilizing activity of the homologous 310–372 ZEB1 fragment was minor (Figure 4a). Treatments of EGFP-ZEB2-transfected cells with lysosomal and proteasomal inhibitors have shown involvement of proteasome in EGFP-ZEB2 destabilization (Figure 4b).

We proposed that the ZEB2 372–437 aa region interacts with the proteins which inhibit protein expression. Therefore, we performed a pull-down experiment followed by mass spectrometric identification of these supposedly important interactions. The EGFP-ZEB2 aa 372–437 construct or wild-type EGFP were expressed in MDA-MB-468 cells and following 16 h proteasomal inhibition, proteins bound to EGFP or EGFP-ZEB2 were purified. Mass spectrometric analysis of the proteins co-purified with the EGFP-ZEB2 aa 372–437 fusion, but not with EGFP control, revealed several proteins belonging to the aminoacyl transfer RNA (tRNA) synthetase complex. Moreover, pull-down experiments have shown that proteins co-immunoprecipitated with EGFP from EGFP-ZEB2-transfected cells were enriched for several subunits of translation elongation factors (Supplementary Table S1). Aminoacyl tRNA synthetase complex is known to co-localize with ribosomes *in vivo*, and they co-sediment with ribosomes in sucrose gradients.^16, 17^

**Figure 5:**
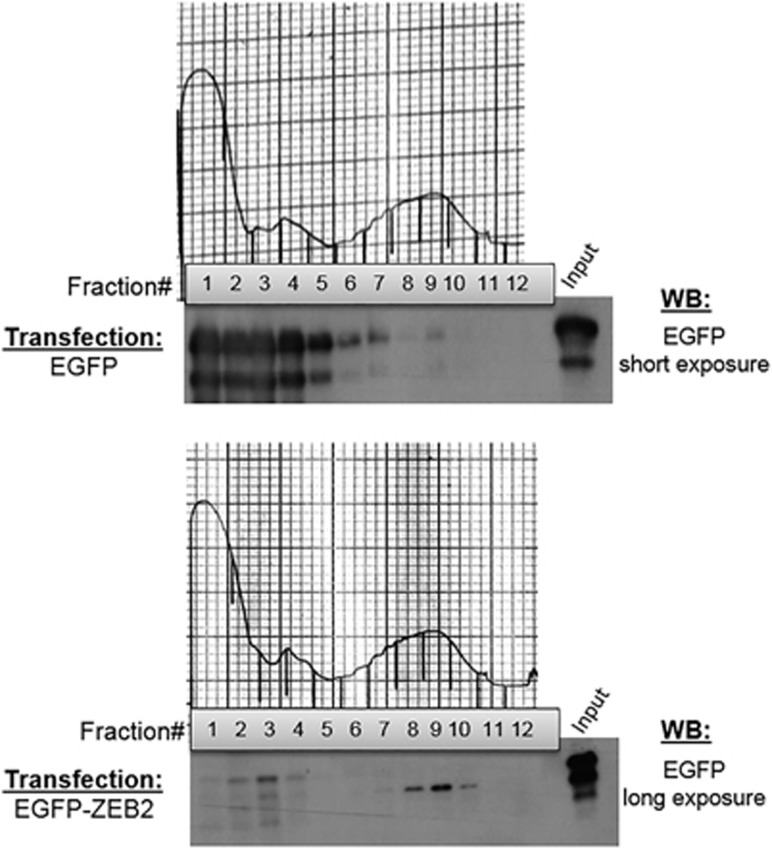
Polysome profiling of EGFP-ZEB2 fusion and control EGFP. MDA-468 cells were transfected with either EGFP-ZEB2 or EGFP-expression vectors; the extracts were prepared 48 h post transfection in a cycloheximide-containing buffer (see Materials and Methods section). Extracts were resolved by sedimentation on 10–50% linear sucrose density gradients and fractionated in 12 fractions. The UV absorbance in each fraction was monitored at 260 nm. To concentrate samples, proteins in each fraction were precipitated using 20% (w/v) trichloroacetic acid, dissolved in laemmli buffer, resolved by 12% SDS-PAGE and analyzed by immunoblotting.

The outcome of these experiments was compatible with the hypothesis that the mechanism underlying reduced expression of EGP-ZEB2 chimaeras involves stalling of elongating ribosomes during translation. When elongation represents the rate-limiting step in translation, one can expect high association of messenger RNA and nascent peptides with polysomes.^18, 19^ To examine this, we performed polysomal profiling of MDA-MB-468 cells expressing EGFP-ZEB2 fusion protein, again using wild-type EGFP as a control. Polysomal fractionation revealed significant enrichment of the EGFP-ZEB2 aa 372–437 fusion protein in the elongating polysomal fractions (fractions 8–10) as compared with EGFP (Figure 5). Of note, the EGFP-ZEB2 372–437 aa fusion protein in the polysomal fractions was detected as a band that migrated faster than EGFP-ZEB2 present in fractions 1-4. Thus, >50% of EGFP-ZEB2 fusion protein was present in polysomal fraction in a form of either nascent or partially degraded polypeptide. In addition, we carried out polysomal fractionation of EGFP- or EGFP-ZEB2 fusion-expressing cells treated with MG132 for 16 h, that is, under conditions we used for pull-down experiments. Prolonged inhibition of proteasomes almost completely eliminated the polysomal fraction. Under these circumstances, control EGFP was almost entirely present in the cytosolic fraction, but a large proportion of the EGFP-ZEB2 protein was found in fraction 4 (80 S monoribosomes) (Supplementary Figure S4). We therefore concluded that 372–437 ZEB2 protein fragment induces association with ribosomes and may therefore determine pausing in translation elongation.

### A stretch of three rare codons reduces expression of ZEB2 in context-dependent manner

**Figure 6:**
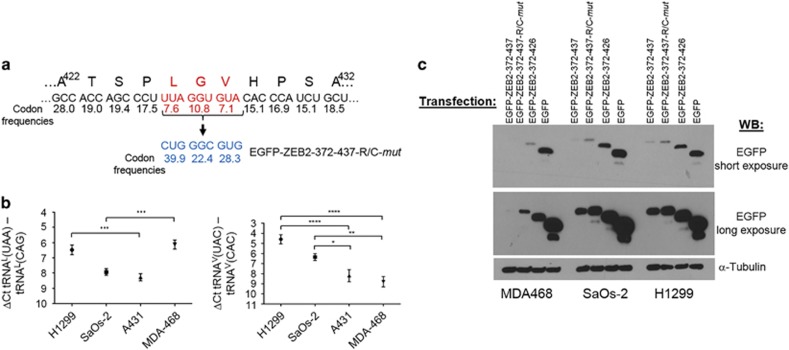
Stretches of rare codons contribute to the translation-reducing activity of the 372–437 aa ZEB2 region. (**a**) 422-432 aa ZEB2 sequence contains a triplet of rare codons, LGV (shown in red). The codon frequencies are indicated. Synonymous codons introduced in the EGFP-ZEB2-372–437- R/Cmut and the full-length EGFP-ZEB2-R/Cmut mutants are shown in blue. (**b**) Relative expression of isoacceptor Leucine and Valine tRNA corresponding to common and rare codons. qPCR analysis was carried out as described in Materials and Methods in triplicate, and ΔCt (rare minus common) are shown as mean ±s.e.m. of three independent experiments (ANOVA). ****P<0.0001; ***P=0.002; **P<0.01; *P<0.05. (**c**) Indicated cell lines were transfected with vectors expressing EGFP, EGFP-ZEB2-372–437, EGFP-ZEB2-372-426 fusion protein, or EGFP-ZEB2-372–437- R/C^mut^ construct, in which a cluster of rare codons was substituted with common codons, as shown in (**b**). Expression of chimeric proteins was analyzed by immunoblotting.

Ribosome pausing during protein synthesis can be inflicted by messenger RNA secondary structure, messenger RNA associated-proteins or codon usage. Examination of the ZEB2 (372–437) ORF using codon frequencies search program (http://www.molbiol.ru/eng/scripts/01_11.html) revealed a single rare codon cluster consisting of three codons coding for leucine, glycine and valine (L^426^G^427^V^428^) (Figure 6a). Noteworthy, this cluster was a part of 426–437 aa sequence that was essential for the inhibiting activity of the 372–437 aa ZEB2 protein fragment (Figure 3b). It is commonly assumed that the concentration of tRNA in the cytosol correlates with the codon frequencies.^20, 21^ However, the ratios in the amounts of individual tRNA varies between different tissues and cell lines. There is no gene for tRNA^G^-ACC in human genome, and the GGU codon is recognized by other tRNA species according to the wobble rules (http://gtrnadb.ucsc.edu). Therefore, we aimed to address whether the expression of *tRNA*^*L*^*-UAA* and *tRNA*^*V*^*-UAC* genes is lower than that of isoacceptor *tRNA* genes corresponding to the common codons, *tRNA*^*L*^*-CAG* and *tRNA*^*V*^*-CAC*. We performed RNAseq analyses of small RNAs expressed in three epithelial cell lines. However, we were not able to identify tRNA^L^-UAA and tRNA^V^-UAC in the data. Detection of individual isoacceptors is not an easy task, because usually they are encoded by several (up to 32) genes exhibiting marked sequence variations (http://gtrnadb.ucsc.edu). We adapted stem–loop qRT-PCR technique for the detection of isoacceptor tRNAs by designing primers homologous to the conserved parts of the genes with forward primers containing anticodon sequences at their 3’-ends (see Materials and Methods). In four cell lines, *tRNA*^*L*^*-UAA* and *tRNA*^*V*^*-UAC* genes were expressed at much lower levels than genes encoding synonymous isoacceptor tRNAs, tRNA^L^-CAG and tRNA^V^-CAC (~60–300- and 30-500-fold difference for *tRNA*^*L*^ and *tRNA*^*V*^ genes, respectively). Remarkably, the ratio of common to rare valine (but not lysine) isoacceptor tRNA expression correlated with the differentiation status of the cell lines. tRNA^V^-UAC corresponding to the rare codon was underrepresented in epithelial cell lines, A431 and MDA-468 (Figure 6b). In the human genome, tRNA^V^-UAC and tRNA^L^-UAA are encoded by six and five genes, respectively, all localized to chromosomes 6, 10, 11 and X (Supplementary Tables S2 &3) (http://gtrnadb.ucsc.edu). We cloned tRNA^V^-UAC and tRNA^L^-UAA tRNAs expressed in MDA-468 and SaOs-2 cell lines, and determined their nucleotide sequence. For cloned tRNA^V^-UAC, all clones (*n*=18) obtained from either cell line contained identical inserts corresponding to two indistinguishable genes on chromosome 11 (Supplementary Table S2). This suggests that the difference in tRNA^V^-UAC levels in MDA-468 and SaOs-2 cells is caused by differential activities of the same gene(s), rather than by the expression of additional genes in SaOs-2 cells. In contrast, two *tRNA*^*L*^*-CAG* genes located on different parts of chromosome 11 were differentially activated in MDA-468 and SaOs-2 cell lines (Supplementary Table S3).

The availability of tRNAs that serve a particular codon is an important factor determining the rate of translation.^22, 23^ In line with the reduced expression of *tRNA*^*L*^*-UAA and tRNA*^*V*^*-UAC* genes, synonymous substitutions of UUA GGU GUA codons by CUG GGC GUG sequence (Figure 6a) strongly enhanced expression level of the ZEB2-EGFP fusion protein in different cell lines with the greatest difference observed in the epithelial carcinoma cell line MDA-468 (Figure 6c).

**Figure 7:**
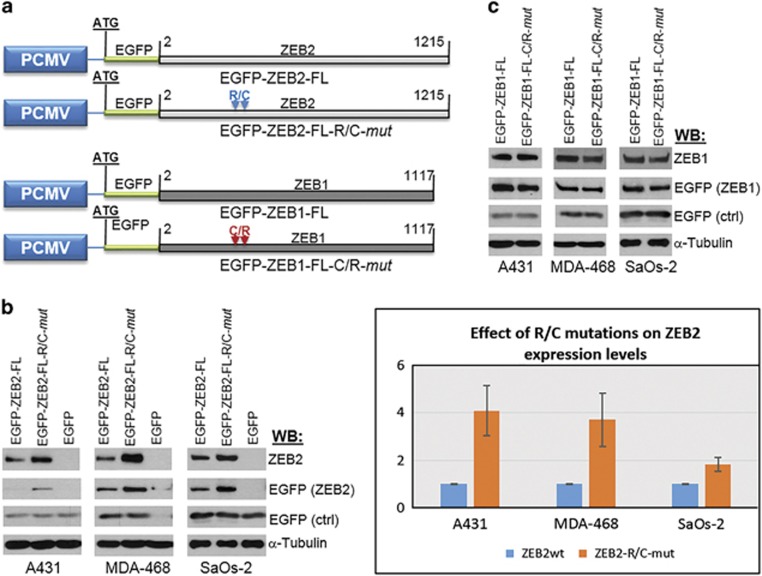
Clusters of rare codons LGV affect protein levels in the context of full-length ZEB2, but not ZEB1 protein sequences. (**a**) A scheme represents EGFP-fused wild-type or mutant ZEB2 or ZEB1. Synonymous rare-to-common codon substitutions are indicated in red, and the replacement of LQA and VQA triplets within ZEB1 with the clusters of rare LGV codons is shown in blue. (**b**) Expression levels of wild-type and mutant ZEB2 protein was analyzed in A431, MDA-468 or SaOs-2 cell lines. Cells were co-transfected with the wild-type EGFP-expression vector, and expression of ZEB proteins was normalized to EGFP levels. The bar chart represents results of four experiments (mean ±s.d.). The results are significant at *P*<0.05 in all cell lines (two-tail *t*-test). (**c**) Incorporation of rare LGV triplets into the ZEB1 sequence does not affect ZEB1 expression levels. Cells were transfected with the vectors expressing wild-type or C/R mutant ZEB1 fused with EGFP (**a**) along with the pEGFP-C1. Expression of ZEB1/EGFP fusions or wild-type EGFP was analyzed by immunoblotting.

Next, we aimed to analyze a role for LGV triplet in the full protein context. Interestingly, ZEB2 contains a second rare codon cluster identical to L^426^G^427^V^428^ and located at the distance of just 10 amino acids C-terminally (L^439^G^440^V^441^). The prospective importance of this motif is evident from the examination of the original truncated mutant series in MDA-468 cells (Supplementary Figure S5), where we detected a sharp increase in fusion protein level following insertion of a stop codon at position 437. Substitution of both rare LGV clusters with common LGV codons evidently increased ZEB2 expression level in MDA-468, A431 and SaOs-2 cells (Figure 7). Next, we carried out the reverse experiment where two triplets located in homologous region of ZEB1, L^361^Q^362^A^363^ (codon frequencies 13.3, 39.3 and 15.9) and V^371^Q^372^A^373^ (codon frequencies 28.9, 11.5 and 26.4), were replaced with LGV rare codons clusters. Importantly, these mutations produced no effect on EGFP-ZEB1 expression (Figure 7c). These data indicate that LGV clusters influence protein expression in appropriate sequence context.

## Discussion

Codon adaptation index (CAI) is a coefficient that ranges from 0 to 1 and characterizes codon usage bias in each particular gene. Higher CAI values indicate a higher proportion of the most common codons. It has been known for decades that in both prokaryotes and eukaryotes highly expressed genes have higher CAIs.^24, 25, 26^ However, the data on impact of codon usage on the efficiency of translation of individual proteins is limited. Recent studies addressed how the codon bias affects the biological features of RAS family members.^27, 28^ This work demonstrated that the difference in the expression levels between KRAS and HRAS is determined by the high proportion of rare codons in *KRAS*. Moreover, differences in CAIs explains the differences in oncogenic potentials of RAS family members and their ability to induce senescence and transformation of murine cells. In addition, these studies identified pairs of genes with high similarity and different content of rare codons. The analysis of 12 of such gene pairs demonstrated that genes with higher CAIs expressed more proteins. Although our data align with the idea that codon bias impact on the regulation of gene expression, we propose that not just the proportion of rare codons in a given gene, but also their distribution throughout the ORF is a factor determining inefficient protein translation. Indeed, CAIs measures for *ZEB1* and *ZEB2* genes equal to 0.70 and 0.76, respectively, and compared with other EMT-TFs, the representation of the rare UUA(L), GGU(G) and GUA(V) codons is exceptionally high in genes encoding ZEB proteins (data not shown). Therefore, the structure of both genes is supposed to be equally adjusted for the translation. However, contrary to this assumption, ZEB2 is translated much less efficiently than ZEB1 because of the presence of triplets of rare codons, LGV, adjacent to the SMAD-binding domain of ZEB2. Whereas LGV triplets are capable of reducing protein translation in the protein context of ZEB2, they are becoming non-functional when incorporated in homologous area of ZEB1.

Both ZEB1 and ZEB2 are stable proteins with half-lives of >5 h. However, fusion with the 372–437 aa ZEB2 protein fragment strongly reduced stability of EGFP, and the experiments with MG132 indicated that EGFP-ZEB2 is a substrate for proteasomal degradation. SKP1-PAM-FBXO45 E3 ligase was shown to ubiquitinate ZEB2 *in vitro* and promote its proteasomal degradation.^14^ Proteasomes are co-localized with polysomes where they assure proper co-translational folding by degrading incorrectly folded nascent peptides.^29^ In some cases, ubiquitination of nascent protein chains is part of co-translational quality control mechanism.^30^ However, ectopic expression of FBXO45, or FBXO45 depletion by RNAi produced no effect on the levels or MG132 sensitivity of EGFP destabilized by the fusion with 372–437 aa ZEB2 protein fragment (data not shown). We applied a JPred program to predict secondary structure of the *N*-terminal part of ZEB2 protein. In all, ~383–437 amino-acid region of ZEB2 is a beta-strand that includes LGV triplet implicated in the structure of this protein domain. Here, we propose a model where a triplet of rare codons induces ribosome stalling and prevents formation of a beta-strand. This results in ubiquitin-independent degradation of the unfolded nascent peptide by a proteasome, which abrogates protein translation.

Apparently, this step of ZEB2 regulation is influenced by several important factors. The pull-down assay with EGFP-ZEB2 fusion revealed an enrichment for the components of the chaperonin complex TRiC (T-complex proteins eta, theta, zeta and epsilon) (data not shown), which are involved in the control of nascent peptide folding. Interaction with TRiC will affect folding and degradation of ZEB2 protein during translation. Another important factor is expression level of tRNA^L^-UAA and tRNA^V^-UAC corresponding to the first and the third codons in LGV triplet, because the availability of these tRNAs must be important for ribosome pausing. In this study, we specifically addressed the expression of rare tRNA^L^-UAA and tRNA^V^-UAC relative to the expression of their common isoacceptor tRNAs (tRNA^L^-CAG and tRNA^V^-CAC). We found that rare isoacceptor tRNAs are underrepresented in epithelial carcinoma cells of different origins (mammary adenocarcinoma MDA-468 and epidermoid squamous carcinoma A431 cell lines) as compared to mesenchymal cells (osteosarcoma SaOs-2 and non-small cell lung cancer H1299 cell lines).

In human genome, five genes positioned on four chromosomes code for the rare isoacceptor tRNA^V^. We found that in two non-related cell lines, MDA-468 and SaOs-2, genes located on the chromosome 11 are active, whereas remaining three genes on chromosomes 6, 10 and X are silent. These observations are in line with the chromatin immunoprecipitation/sequencing data demonstrating that PolIII occupies only a fraction of all tRNA gene promoters *in vivo*.^31, 32^ We also found that the abundance of the rare tRNA isoacceptors tRNA^V^-UAC and tRNA^L^-UAA is different in epithelial and mesenchymal cells. This is consistent with the findings that tRNA expression patterns are tissue-specific and differ between proliferating and differentiating cells.^33, 34^ Though transcriptional regulation of individual *tRNA* genes remains largely uncharacterized, it has been demonstrated that PolII and PolIII are present at the same genomic loci in open chromatin areas. Therefore, expression pattern of protein-coding genes seems to determine the spectrum of active *tRNA* and other PolIII-transcribed genes.^35^ Global differences in gene expression between epithelial and mesenchymal cells may explain our observation that epithelial and mesenchymal cells express individual *tRNA* genes at different levels.

ZEB2 protein may have an oncosuppressive role by inhibiting cell cycle progression and maintaining cell differentiation.^6, 7, 9, 36^ In contrast, ZEB1 drives EMT pathways, which cooperate with classical oncogenes in malignant transformation.^9, 37^ Therefore, the physiological outcomes of EMT programs activated by ZEB proteins in tumor cells are often different. Our data propose that some previously unrecognized factors, such as availability of certain tRNA isoacceptors or TRiC, may generate permissive conditions for ZEB2 expression and determine the configuration of EMT pathways. This hypothesis is in line with recent research highlighting role of individual tRNAs in cancer metastasis.^38^

## Materials and methods

### Cell lines

Cell lines were obtained either from Wellcome Trust Functional Genomics Cell Bank (St George’s, University of London, UK) or from the ATCC (American Type Culture Collection) or and cultured according to the ATCC recommendations. Authentication of cell lines was carried out by Short Tandem Repeat Profiling.

### Plasmid construction and transfections

pZ/EG-HA-ZEB1 and pZ/EG-HA-ZEB2 expression vectors enabled Cre-mediated ZEB protein expression with concomitant activation of EGFP. To generate these constructs, we PCR-amplified and subcloned the ZEB1 or ZEB2 open reading frames^9^ into BglII and XhoI or BglII and XhoI/SalI restriction sites of the pZ/EG vector.^39^ *N*-terminal HA-tag flanked by the Kozak sequence derived from the *ZEB1* gene (GAGGATC) was incorporated in both constructs.

To generate pEGFP-ZEB1 and pEGFP-ZEB2, sequences coding for ZEB1 310–372 aa or ZEB2 372–437 aa sequences were inserted into BglII and KpnI sites of pEGFP-C1 (Clontech, Mountain View, CA, USA in frame with EGFP. To construct pZEB1-EGFP and pZEB2-EGFP the same sequences were cloned in BglII and XhoI sites in frame with EGFP in the pEGFP-N2 vector (Clontech).

To generate vectors expressing full-length ZEB proteins fused with EGFP, their open reading frames were cloned in pEGFP-C1 vector using BglII and KpnI restriction sites.

To introduce mutations in the expression constructs, standard site-directed mutagenesis was carried out using Quick Change kit from Stratagene (Agilent Technologies, Santa Clara, CA, USA). All plasmids were verified by sequencing (GATC Biotech, Konstanz, Germany).

For transfections by electroporation, cells were treated with a single pulse of 250 V and 250 Fd by using the Gene Pulser Xcell electroporation system (BioRad Laboratories, Hercules, CA, USA). Some cell lines were transfected using JetPRIME DNA transfection reagent (Polyplus-Transfection, Illkirch France).

### Immunoblotting

Immunoblotting was carried out according to standard procedures.^12^ The following primary antibodies were used in this study: anti-EGFP (ImmunoKontact, UK; catalog number 210-PS-1GFP), anti-ZEB1 (Santa Cruz Biotechnology, Dallas, TX, USA; sc-25388); anti-SNAIL2 and anti-Cyclin D1 (Cell Signaling Technology, Danvers, USA; 9585 and 2978) anti-ZEB2 (in-house^12^); anti-HA and anti-α-Tubulin antibodies from Sigma-Aldrich (St Louis, MO, USA; H3663 and T5168); anti-TWIST1 antibody from Abcam (Cambridge, MA, USA; AB50887); and anti-E-cadherin (BD Bioscience, San Jose, USA; 610181).

### Polysome profiling

Polysome isolation was performed as described previously.^40^ In brief, cells were incubated with 100 μg/ml cycloheximide for 3 min prior harvesting. Cell pellets were lysed with the polysome lysis buffer (0.3M NaCl; 15 mM MgCl2; 15 mM Tris-HCl, pH 7.5) containing 1% v/v Triton-X. The nuclei were precipitated by centrifugation at 13000 rpm (4 °C, 1 min), and the supernatant was loaded onto the top of the 10–50% linear sucrose density gradients. Next, the samples were separated by centrifugation in Sorvall WX Ultra Series Centrifuge (Thermo Fisher Scientific, Waltham, MA, USA) at 38 000 rpm for 2 h at 4 °C. After centrifugation, the samples were fractionated and absorbance at 254 nm was monitored using UA-6 UV/VIS Detector machine (Teledyne Isco, Lincoln, NE, USA). The samples were collected into 12 fractions 1 ml each. Proteins were precipitated using 20% (w/v) trichloroacetic acid and analyzed by immunoblotting.

### Pull-down and mass spectrometry

Transfected cells were grown on two 150 mm petri dishes. After 48 h of transfection they were harvested and lysed in NP-40 lysis buffer (10 mM Tris-Cl, pH 7.4; 10 mM NaCl; 3 mM MgCl2; 0.5% NP-40) containing protease inhibitor cocktail (Roche Diagnostics Ltd, Burgess Hill, UK). Pull-down was carried out using GFP-Trap agarose beads (ChromoTek GmbH, Martinsried, Germany) according to the manufacturer’s protocol. Proteins were eluted from the beads in laemmli buffer, applied to SDS-PAGE, digested with trypsin (Promega, Southampton, UK) and identified by LC-MS/MS using an LTQ-Orbitrap Velos mass spectrometer (Thermo Fisher Scientific) as recently described (Tsimokha *et al.*, 2017).^41^

### tRNA analysis

We modified stem–loop quantitative PCR (qPCR) method for quantitative analysis of the expression of individual tRNAs. Total RNA was isolated from MDA-468 and SaOs-2 cells using RNeasy RNA isolation kit (Qiagen, Germantown, USA) and applied for the complementary DNA synthesis using stem–loop reverse transcription primers. Whereas two separate primers were designed for the reverse transcription of abundant and rare valine tRNAs, *tRNA*^*V*^*-CAC* and *tRNA*^V^*-TAC*, one common primer was used for the synthesis complementary DNA corresponding to both rare and abundant leucine tRNAs (Supplementary Table S4). Another stem–loop primer was designed for the reverse transcription of 18 S rRNA used as an internal control. A total of 100 ng of RNA were mixed with the stem–loop primers, denatured at 75 °C for 5 min, allowed to anneal on ice, and subjected to reverse transcription reaction using the AMV reverse transcriptase (Promega Corp., Madison, WI, USA). The reaction was carried out for 30 min at 16 °C followed by the additional 30 min incubation at 42 °C. For qPCR analysis, we designed a universal reverse primer corresponding to the 17–41 nt sequence of stem–loop primers; forward primers were unique and included anticodon sequences at the 3’-ends (Supplementary Table S5). qPCR reactions were carried out using Fast SYBR Green Master Mix (Applied Biosystems, Warrington, UK) in 40 cycles with the annealing/extension temperature 60 °C.

To identify *tRNA* genes expressed in MDA-468 and SaOs-2 cells, the products of the PCR reaction were cloned in pDrive vector (Qiagen) and DNA samples from 10 individual clones per each PCR product were subjected to the sequencing (GATC Biotech). Design of the primers allowed us to distinguish between most of the *tRNA* paralogues, because of single nucleotide differences between them (except for identical *tRNA*^V^*-TAC-1-1* and *tRNA*^V^*-TAC-1-2* genes).

### Statistical analysis

Statistical analysis of the data was carried out using Student’s *t*-test (two-tailed, unpaired) or one-way analysis of variance.
