# Supplementary material for: Short stretches of rare codons regulate translation of the transcription factor ZEB2 in cancer cells
Source: Oncogene. 2017 Aug 7;36(47):6640–8. doi: 10.1038/onc.2017.273 (PMC5681250; doi:10.1038/onc.2017.273)
Supplement: Supplementary Figures and Tables [file onc2017273x1.pdf]

## SUPPLEMENTARY FIGURES AND TABLES

# Short stretches of rare codons regulate translation of the transcription factor ZEB2 in cancer cells

Wan Makhtar WR[1, 4, 6], Browne G[1, 6], Karountzos A[1], Stevens C[1], Alghamdi Y[1], Bottrill AR[3], Mistry S[3], Smith E[4], Bushel M[4], Pringle JH[1], Sayan AE[5], Tulchinsky E[1].

[1]Department of Cancer Studies, University of Leicester, Leicester, UK
[2]Current address: School of Medical Sciences, University Sains Malaysia, MALAYSIA
[3]Protein and Nucleic Acid Chemistry Laboratory (PNACL), University of Leicester, UK
[4]MRC Toxicology Unit, Leicester, UK
[5]Cancer Sciences, University of Southampton, UK
[6]Co-first authors

**Figure S1**

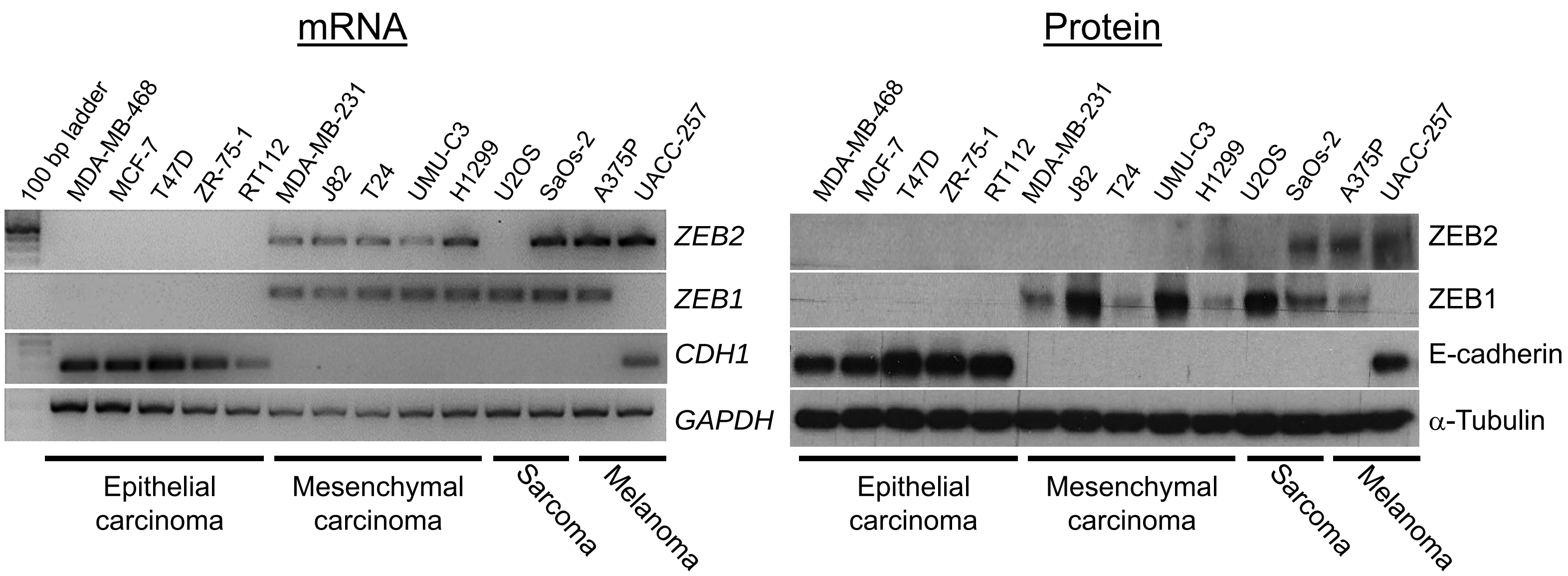


**Figure S1**. Expression levels of ZEB1/2 mRNA (RT-PCR, left panel) and protein (Western blotting, right panel) in a series of cell lines. Expression of the *CDH1* gene and E-cadherin protein specifies epithelial cell lines and a melanoma cell line, UACC-257.

# Figure S2

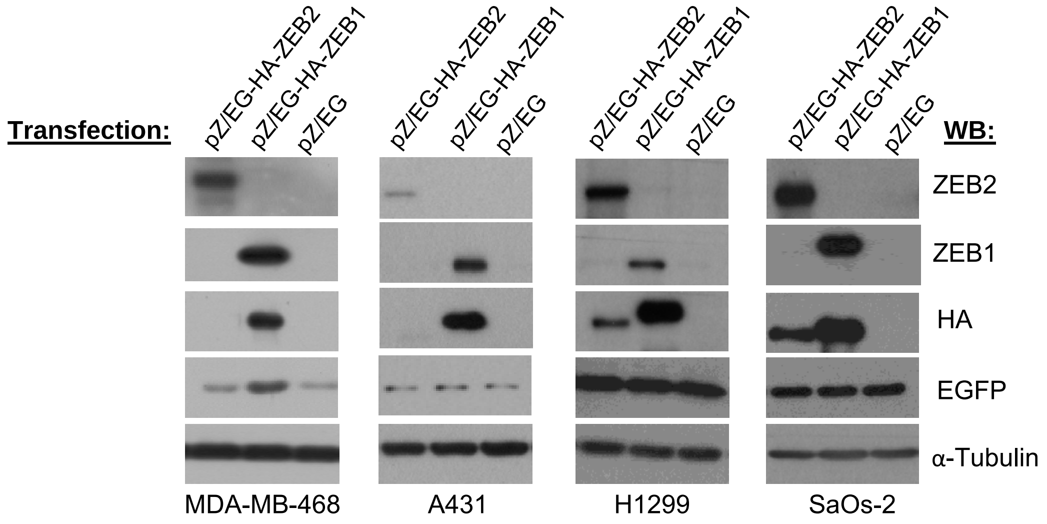


**Figure S2.** Indicated cell lines were transfected with pZ/EG vectors co-expressing ZEB1 or ZEB2 with EGFP, or with a wild-type vector expressing EGFP only. 1 μg of pCre recombinase expression vector DNA was added to each transfection. Protein expression was analyzed by immunoblotting as shown.

# Figure S3

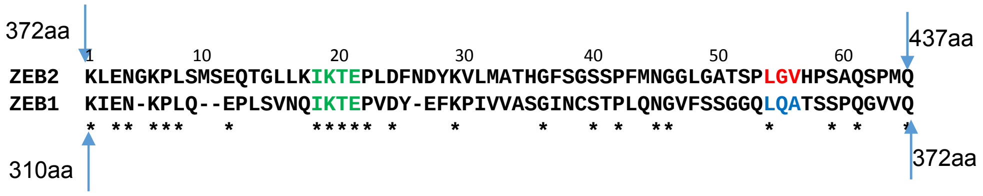


**Figure S3**. Alignment of the ZEB2 372-437 aa sequence and its homologous fragment in ZEB1. A triplet of rare codons LGV and corresponding sequence in ZEB1 LQA are indicated in red and blue respectively. Sumoylation sites are shown in green.

# Figure S4

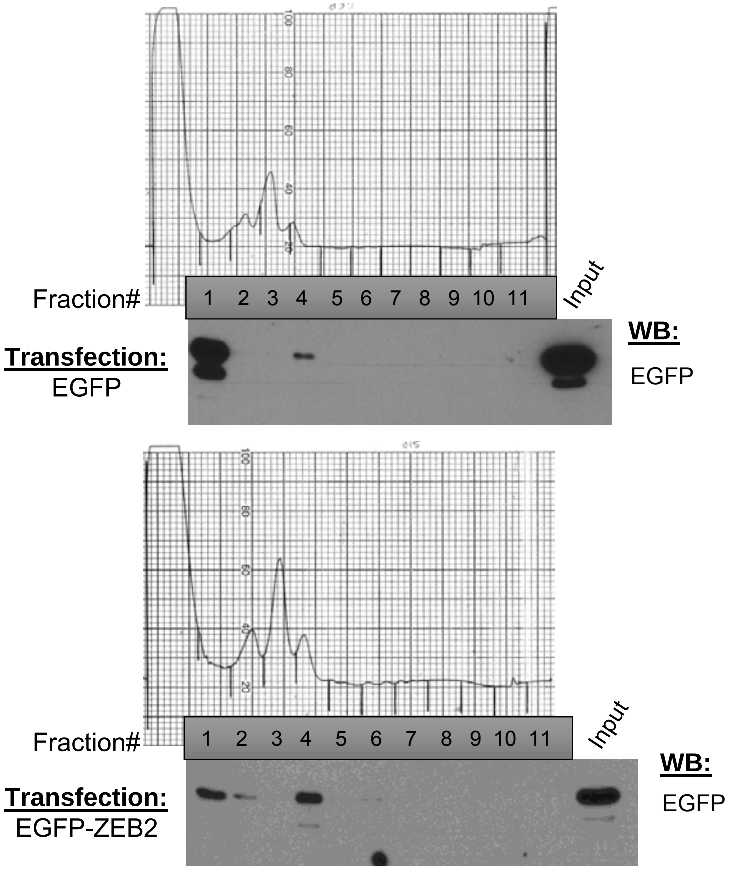


**Figure S4**. Polysome profiling of EGFP-ZEB2 fusion and control EGFP in MDA-468 cells treated with MG132. MDA-468 cells were transfected with either EGFP-ZEB2- or EGFP-expression vectors. The transfected extracts were treated with MG132 for 16 hours prior harvesting. The cells were lysed as described in Materials and Methods section. Extracts were resolved by sedimentation on 10–50% linear sucrose density gradients and fractionated in 11 fractions. The UV absorbance in each fraction was monitored at 260 nm. To concentrate samples, proteins in each fraction were precipitated as described and analyzed by immunoblotting.

## Figure S5

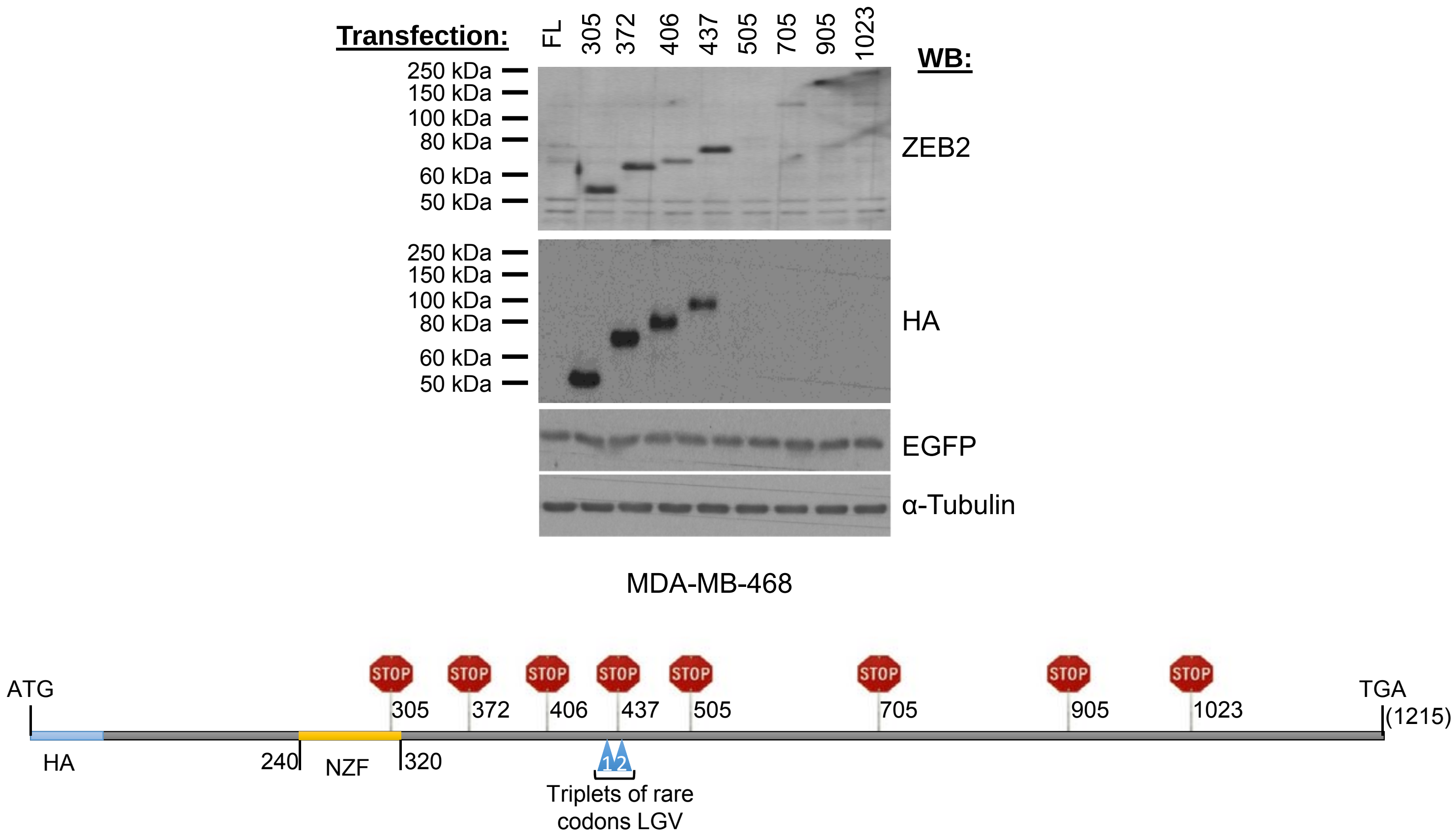


**Figure S5**. A431 cells were transiently transfected with the series of constructs producing truncated ZEB2 proteins along with an EGFP-expression vector. Expression levels of EGFP, full-length (FL) or truncated ZEB2 protein was analyzed by immunoblotting. A scheme illustrates positions of "stop" and clusters of rare codons.

**Table S1.**

**Proteins associated with translation machinery interact with EGFP-ZEB2, but not EGFP. Complexes were isolated from MDA-468 cells, and proteins identified by mass spectrometry**

| Protein | Identified peptides | Expect | Score |
|---|---|---|---|
| Elongation factor 1-alpha 1 | R.YEEIVK.E<br>K.EVSTYIK.K<br>K.QLIVGVNK.M<br>R.TIEKFEK.E<br>K.EVSTYIKK.I<br>R.LPLQDVYK.I<br>K.IGGIGTVPVGR.V<br>K.STTTGHLIYK.C | 0.0065<br>0.012<br>0.019<br>0.0058<br>0.079<br>0.00051<br>1.1e-005<br>0.0035 | 30<br>28<br>26<br>31<br>20<br>44<br>57<br>36 |
| Elongation factor 1-gamma | K.AKDPFAHLPK.S<br>K.STFVLDEFKR.K<br>R.AVLGEVKLCEK.M<br>M.AAGTLYTYPENWR.A<br>K.AAAPAPEEEMDECEQALAAEPK.A | 0.0001<br>1.1e-005<br>1.5e-005<br>0.046<br>1.5 | 49<br>59<br>59<br>22<br>3 |
| Elongation factor 1-delta | K.LVPVGYGIR.K<br>R.IASLEVENQSLR.G | 0.0031<br>1.7e-006 | 36<br>69 |
| Elongation factor 1-beta | R.SIQADGLVWGSSK.L | 2.2e-005 | 58 |
| Elongation factor 2 | M.VNFTVDQIR.A | 5.5e-006 | 64 |
| Bifunctional aminoacyl-tRNA synthetase | R.LLSVNIR.V<br>K.YYTLFGR.S | 0.0032<br>0.0035 | 37<br>37 |
| Glutaminyl-tRNA synthetase | R.LAWGQPVGLR.H<br>K.AINFNFGYAK.A | 0.00082<br>0.42 | 39<br>15 |
| Lysyl-tRNA synthetase | K.ILDDICVAK.A<br>M.AAVQAAEVKVDGSEPK.L | 0.12<br>4.3e-007 | 21<br>75 |
| Aminoacyl tRNA synthase complex-interacting multifunctional protein 2 | R.VLSTVHTHSSVK.S<br>R.SCENLAPFNTALK.L | 0.0053<br>0.00013 | 32<br>48 |
| Aspartyl-tRNA synthetase | R.GEEILSGAQR.I | 0.018 | 29 |

## Table S2

**Profiling of *tRNA$^{V}$* in MDA-468 and SaOs-2 cell lines. Numbers of clones corresponding to the individual genes are shown**

| tRNA gene | Locus | MDA-468 | SaOs-2 |
|---|---|---|---|
| *tRNA-Val-TAC-1-1* | chr11:59318102-59318174 | 9 | 9 |
| *tRNA-Val-TAC-1-2* | chrX:18693029-18693101 | 0 | 0 |
| *tRNA-Val-TAC-2-1* | chr11:59318460-59318532 | - | - |
| *tRNA-Val-TAC-3-1* | chr10:5895674-5895746 | 0 | 0 |
| *tRNA-Val-TAC-4-1* | chr6:27258405-27258477 | 0 | 0 |
| *tRNA-Val-TAC-4-1* | chr6:27258405-27258477 | 0 | 0 |

## Table S3

**Profiling of *tRNA$^{L}$* in MDA-468 and SaOs-2 cell lines. Numbers of clones corresponding to the individual genes are shown**

| tRNA gene | Locus | MDA-468 | SaOs-2 |
|---|---|---|---|
| *tRNA-Leu-TAA-1-1* | chr6:144537684-144537766 | 2 | 5 |
| *tRNA-Leu-TAA-3-1* | chr11:59319228-59319310 | 1 | 2 |
| *tRNA-Leu-TAA-2-1* | chr6:27688898-27688980 | 0 | 0 |
| *tRNA-Leu-TAA-4-1* | chr6:27198334-27198416 | 5 | 1 |
| *tRNA-Leu-TAA-11-12* | chr11:113432995-113433078 | 0 | 0 |

# Tables S4 & S5

**Table S4. Sequences of stem-loop primers used for the reverse transcription reactions. Sequences homologous to the *tRNA* or *18S rRNA* genes are shown in red.**

| Gene | Stem-loop primer sequence |
|---|---|
| *tRNA$^{V}$-CAC* | GTCGTATCCAGAATTTGTTGCAACGAACAGGTCTGGATACGACT**TTCYGCCCG** |
| *tRNA$^{V}$-TAC* | GTCGTATCCAGAATTTGTTGCAACGAACAGGTCTGGATACGACT**TTCCACTGG** |
| *tRNA$^{L}$-TAA* & *tRNA$^{L}$-CAG* | GTCGTATCCAGAATTTGTTGCAACGAACAGGTCTGGATACGACT**AACCCACGC** |
| *18S rRNA* | GTCGTATCCAGAATTTGTTGCAACGAACAGGTCTGGATACGA**CTAATGATC** |

**Table S5. Sequences of the primers used in qPCR reactions. Anticodons are shown in red.**

| Gene | Sequence |
|---|---|
| *tRNA$^{V}$-CAC* | TAGTGGTTATCACGTTCGCCT**CAC** |
| *tRNA$^{V}$-TAC* | TAGTGGTTATCACGTCTGCTT**TAC** |
| *tRNA$^{L}$-CAG* | GGTCTAAGGCGCTGCGTT**CAG** |
| *tRNA$^{L}$-TAA* | GAGTGGATAAGGCGTTGGACT**TAA** |
| *18S rRNA* | GTAACAAGGTTTCCGTAGGTGA |
| Reverse primer (universal) | GTTGCAACGAACAGGTCTGGATACG |
